# Selections from Foreign Periodicals

**Published:** 1887-04

**Authors:** 


A. W. Clement, V. S.,

*Montreal Veterinary College.*

### Selections from Foreign Periodicals.

The Contagium of Influenza Pectoralis. By Professor Schutz. Tageblatt der 59 Versamlung. Deutscher Natur-forscher und Arzte in Berlin, September 21, 1886.

Anatomically there are two forms of influenza seen in horses. In one there are yellow necrotic spots in the lung tissue, both in the substance of the lung and just beneath the pleura, in which lies the seat of the contagium. From the oldest spots an extension of the disease can take place to the neighboring parenchyma, and also to the pleura (spreading pleuritis). The typical pleuritis of influenza is brought about by the softening of a necrosed spot and perforation of the pleura.

The second form presents no such spots, but there is diffuse hepatization. The first form is a pneumonia multiplex mortificans, very much resembling gangrene; the second a pneumonia simplex like croupous pneumonia of man.

Examination of the necrotic spots showed them to be filled with special bacteria of oval form, mostly two together and with longitudinal fission in the broken spots, but they were sparingly distributed in the immediate neighborhood.

Artificial cultures grow best in gelatine, in the form of kernels in the neighborhood of the inoculation, only under the surface, and without liquefying the gelatine. The bacteria are identical in form and growth with those found in the lung. Mice inoculated die of septicæmia (splenitis hemorrhagia and fatty liver prominent). The bacteria are plentiful in the blood and are here surrounded by an uncolored place. Direct inoculation of cultures into the lungs killed three horses after an illness of eleven days, each time by mortifying pneumonia of the infection sort, with perforating pleuritis. An inhalation experiment on one horse did not cause death; it recovered, and showed when it died, a long time after, chronic pneumonia. This experiment will be carried out further. By the second diffuse form of pneumonia the bacteria were found in the hepatized parts. Simple and mortifying pneumonia are etiologically the same process; the contamination is continued from the spot of infection, and through diffusion becomes general. As the croup has a fibrous and a diptheritic form, so is the pneumonia of every virulence of contagion—at one time a mortifying and at another time only a fibrous inflammation. Therefore, we find in horses only one kind of pneumonia, but which anatomically and clinically show every variety of virulence of the contagium—at one time sporadic, at another epidemic,

A. W. CLEMENT, V. S.

THE CONTAGIUM OF PLEURO-PNEUMONIA. By Drs. J. Poels and W. Nolen, Rotterdam. The *Veterinarian*, March, 1887.

The outcome of the investigations and experiments may be stated as follows: A specific micrococcus always occurs in the lungs of animals affected with pleuro-pneumonia, the morphological and pathological characteristics of which are described. This micrococcus is not present in the lungs of healthy animals. Pure cultivations of these micrococci injected into the lungs of rabbits, guinea-pigs and dogs are able to produce pneumonic changes in these animals, while similar injections of central fluids have no result. Pneumonic changes are also produced by the injection of these micrococci into the trachea of dogs, and as a consequence of their inhalation by mice. Injection of the micrococci, obtained by pure cultivation, into the lungs of a bovine animal caused extensive pneumonic changes within a period of seven days. Bovine animals may be successfully inoculated with the pure cultivation above mentioned. This micrococcus is also always present in the sores resulting from ordinary inoculation. The micrococcus is not present in the malignant inflammatory swellings which often occur in inoculation.

If we now review the results of these investigations, not only each one by itself, but looking at them at the same time in their connection with each other—namely, that the coccus we have described is always found in great numbers in the exudation products of lungs affected with pleuro-pneumonia, but not in healthy lungs; that the same micrococcus is always found in the healthy reaction succeeding inoculation, and, on being developed in pure cultivations, can be successfully used for the purpose of inoculation; and, lastly, that the coccus can in a few days produce extensive pneumonic changes in a bovine animal, we think we are justified in concluding this coccus is the contagium of pleuro-pneumonia. W. A. C.

